# Psychosocial Development in 5-Year-Old Children With Hearing Loss Using Hearing Aids or Cochlear Implants

**DOI:** 10.1177/2331216517710373

**Published:** 2017-07-28

**Authors:** Cara L. Wong, Teresa Y. C. Ching, Linda Cupples, Laura Button, Greg Leigh, Vivienne Marnane, Jessica Whitfield, Miriam Gunnourie, Louise Martin

**Affiliations:** 1 National Acoustics Laboratories (NAL), Macquarie University, NSW, Australia; 2 HEARing CRC, The University of Melbourne, Carlton, Australia; 3 Macquarie University, NSW, Australia; 4 Royal Institute for Deaf and Blind Children (RIDBC), Sydney, Australia

**Keywords:** deaf or hard of hearing, hearing aids, cochlear implant, pediatric, psychosocial, social skills, language, functional communication skills, Parents’ Evaluation of Aural/oral performance of Children, Strengths and Difficulties Questionnaire, Child Development Inventory

## Abstract

This article reports on the psychosocial development and factors influencing outcomes of 5-year-old children with cochlear implants (CIs) or hearing aids (HAs). It further examines differences between children with CIs and HAs with similar levels of hearing loss. Data were collected as part of the Longitudinal Outcomes of Children with Hearing Impairment study—a prospective, population-based study. Parents/caregivers of children completed the Strengths and Difficulties Questionnaire (*n* = 333), the Social Skills subscale from the Child Development Inventory (*n* = 317), and questionnaires on functional auditory behavior (Parents’ Evaluation of Aural/oral performance of Children), and demographics. Children completed assessments of nonverbal cognitive ability (Wechsler Non-verbal Scale of Ability) and language (Preschool Language Scale - fourth edition). On average, parent-rated Strengths and Difficulties Questionnaire scores on emotional or behavioral difficulties were within 1 *SD* of the normative mean; however, Child Development Inventory scores on social skills were more than 1 *SD* below the norm. Children with severe-to-profound hearing losses using HAs had significantly more behavioral problems than children with CIs. Regression analyses showed that non-verbal cognitive ability, language, and functional auditory behavior were significantly associated with psychosocial outcomes for children with HAs, whereas outcomes for children with CIs were associated with functional auditory behavior and the presence of additional disabilities. Age at hearing intervention, severity of hearing loss, and communication mode were not associated with outcomes. The results suggest that even children who develop good language ability with the help of a HA or CI may have psychosocial problems if they exhibit difficulties with listening and communicating in everyday environments. The findings have implications for developing interventions for young children with hearing loss.

## Introduction

Hearing loss, the most common congenital sensory impairment, can have lifelong developmental consequences on communication, language, social, and academic functioning. The literature has reported that, on average, children who are deaf or hard of hearing (DHH) have higher rates of psychosocial problems including emotional disorders (e.g., anxiety and depression), behavioral problems (e.g., hyperactivity and conduct problems), and social difficulties compared with their hearing peers (Fellinger, Holzinger, Sattel, & Laucht, 2008; Hindley, 2005; Mejstad, Heiling, & Svedin, 2009; Netten et al., 2015). These problems have typically been associated with delays in language and communication abilities (Barker et al., 2009; Stevenson, McCann, Watkin, Worsfold, & Kennedy, 2010). Although recent advances in hearing device technology and universal newborn hearing screening have led to improvements in language development, there has been little up-to-date research to conclude whether these advancements have led to improvements in psychosocial functioning (Moeller, 2007; Sorkin, Gates-Ulanet, & Mellon, 2015).

A recent meta-analysis of 45 studies investigated psychosocial difficulties in DHH children or adolescents compared with hearing controls (Stevenson, Kreppner, Pimperton, Worsfold, & Kennedy, 2015). The majority of studies reviewed did not provide information about the hearing device used, or did not report outcomes separately for children with cochlear implants (CIs) or hearing aids (HAs). In general, there appear to be more studies that focus specifically on the psychosocial outcomes of children with CIs, but very few that include only children with HAs, particularly those with milder hearing losses. To address this imbalance, this article examines outcomes and potential mediating factors separately for DHH children using CIs or HAs. The aim is to enhance understanding of how hearing device can influence psychosocial outcomes, with the ultimate goal of informing evidence-based interventions.

### Psychosocial Outcomes of Children With CIs

Cochlear implantation has now become the dominant choice of hearing device for children with profound hearing loss (Punch & Hyde, 2011a). Unlike HAs, which amplify sounds, CIs provide direct electrical stimulation to the user’s auditory nerve (Bat-Chava, Martin, & Kosciw, 2005; Svirsky, Robbins, Kirk, Pisoni, & Miyamoto, 2000). A number of studies have demonstrated the benefits of cochlear implantation for oral language development and integration into mainstream schools (Ching et al., 2009; Geers, Nicholas, & Sedey, 2003). Others have reported that the language and cognitive development of young children with CIs does not differ significantly from that of hearing children (Khan, Edwards, & Langdon, 2005; Svirsky et al., 2000). The enhancement of oral language skills is likely to have positive effects on psychosocial adjustment.

Accordingly, studies looking at parent perceptions of psychosocial function from pre- to postimplantation have found improved quality of life (QoL), self-esteem, and peer relationships (Bat-Chava & Deignan, 2001; Bat-Chava et al., 2005; Leigh, Maxwell-McCaw, Bat-Chava, & Christiansen, 2009; Nicholas & Geers, 2003). A number of studies focusing on the outcomes of children with CIs have found comparable scores to typically developing children on the Strengths and Difficulties Questionnaire (SDQ), a screen of emotional, behavioral, and peer problems (Anmyr, Larsson, Olsson, & Freijd, 2012; Huber & Kipman, 2011). However, these studies have typically used small sample sizes (*n* = 22–35) and older children or adolescents (ages 9 to 17 years; Anmyr et al., 2012; Huber & Kipman, 2011; Leigh et al., 2009; Nicholas & Geers, 2003).

Furthermore, not all studies have reported positive effects of CIs on psychosocial development (Dammeyer, 2010; Huber, 2005; Huber et al., 2015; Peterson, 2004; Pulsifer, Salorio, & Niparko, 2003), and residual difficulties have been reported postimplantation due to delays in oral communication, difficulties in group situations, and attitudes of hearing peers (Bat-Chava & Deignan, 2001; Punch & Hyde, 2011b). Some researchers have also reported that use of CI is not linked directly to improved social competence but influences it via improved communication, increased identification with the hearing world, and mainstream schooling (Bat-Chava & Deignan, 2001; Leigh et al., 2009).

Although it is still unclear whether children with CIs are comparable to their hearing peers in psychosocial development, there is evidence that they have fewer psychosocial problems when compared with DHH children *without* CIs (Bat-Chava et al., 2005; Theunissen et al., 2012; Theunissen et al., 2014a). For example, when compared with children who have moderate to profound hearing loss and use HAs, children with CIs were reported to have lower levels of general anxiety, social anxiety, and behavioral problems (including aggression, attention, and conduct disorders) and were rated as being more similar to their hearing peers (Theunissen et al., 2012; Theunissen et al., 2014a). The authors suggested that the advantage of a CI may lie in its restoring auditory input and lowering social barriers but also that children with CIs may receive more counselling and rehabilitation than those with HAs.

After reviewing the literature, Dammeyer (2010) and Theunissen et al. (2014c) concluded that the psychosocial well-being of DHH children with CIs lay somewhere between that of DHH children without CIs and that of hearing children. However, the majority of studies investigating the psychosocial outcomes of children with CIs specifically are limited by small sample sizes and do not include relevant control groups. Many studies have not considered the possible influence of children’s chronological age, intelligence, age at implantation, duration of implant use, communication mode, oral abilities, additional disabilities, and family background (Dammeyer, 2010; Stevenson et al., 2015). Without controlling for these potential mediating variables, the effect of hearing device on psychosocial outcomes is difficult to establish.

### Factors Influencing Outcomes in Children With CIs

Language and communication ability have long been linked to psychosocial development in both typically developing (Benner, Nelson, & Epstein, 2002; Petersen et al., 2013) and DHH children (Barker et al., 2009; Stevenson et al., 2010). In the hearing population, clinically significant language deficits have been found in approximately three out of four children with diagnosed emotional or behavioral disorders (Benner et al., 2002). Conversely, children with language disorders have a higher incidence of social, emotional, and behavioral problems (Conti-Ramsden & Botting, 2008; St Clair, Pickles, Durkin, & Conti-Ramsden, 2011). Similarly, studies investigating children with CIs have found that better language skills (including speech understanding, speech production, vocabulary, and syntactic proficiency) are significantly associated with better psychosocial outcomes (Percy-Smith, Hedegaard Jensen, et al., 2008; Remmel & Peters, 2009). Notwithstanding this association, evidence has also shown that DHH children are still at higher risk of psychosocial problems such as low self-esteem and empathy after controlling for language ability (Netten et al., 2015; Theunissen et al., 2014b); however, these studies have not looked separately at children with CIs and HAs.

An important consideration relating to successful communication and social interactions in DHH children is their ability to use their devices to listen effectively and communicate in everyday environments, hereon referred to as “functional auditory behavior.” Compromised ability to hear speech or detect subtle cues in conversation can negatively affect social interactions (Batten, Oakes, & Alexander, 2014) and may result in particular difficulties for children with CIs in noisy environments such as the playground or in groups of people (Punch & Hyde, 2011b). Consistent with this view, Huber and Kipman (2011) reported that adolescents with CIs who had good speech perception ability in both quiet and noisy environments were more likely to have SDQ scores within the normal range. Further evidence comes from a recent study of younger DHH children with either CIs or HAs (Leigh et al., 2015). Leigh et al. (2015) reported that children whose parents rated them as being able to listen and communicate well in a range of different quiet and noisy environments, as measured on the Parent Evaluation of Aural/Oral Performance of Children (PEACH) scale, also had more highly developed social skills on the Child Development Inventory (CDI).

For children with CIs, a number of studies have found that younger age at implantation or longer duration of implant use was associated with improved social relationships, communication, self-esteem, and QoL (Bat-Chava & Deignan, 2001; Martin, Bat-Chava, Lalwani, & Waltzman, 2010; Percy-Smith, Cayé-Thomasen, Gudman, Jensen, & Thomsen, 2008; Theunissen et al., 2014b; Yoshinaga-Itano, 2001). Theunissen et al. (2012) reported that children who were implanted earlier and had a longer duration of CI use had less generalized and social anxiety. However, associations between earlier age at implantation and psychosocial outcomes have not been found consistently (Nicholas & Geers, 2003; Percy-Smith, Hedegaard Jensen, et al., 2008).

There are a number of other child- and family-related demographic and social factors that may directly or indirectly impact on psychosocial outcomes (as well as developmental outcomes in general) and consequently should be controlled for when examining the effects of hearing device on psychosocial outcomes. Child-related factors include the presence of additional disabilities (Cupples et al., 2014; Dammeyer, 2010) and non-verbal cognitive ability (Geers & Moog, 1987; Nicholas & Geers, 2003). Family-related factors include child’s communication mode at home (Percy-Smith, Hedegaard, Jensen, et al., 2008), socioeconomic status (SES; Geers, 2002), and maternal education (Ching, Dillon, et al., 2013). Although there is evidence that child-related factors impact directly on psychosocial outcomes, the evidence is less clear for these family-related factors, which may only be indirectly related through their influence on language development and communication (Wong et al., 2016).

### Outcomes of Children With HAs

There are very few studies focusing specifically on the psychosocial outcomes of children with mild to moderate hearing losses who use HAs. Not surprisingly, research has shown that children with severe to profound losses who use HAs (e.g., preimplant CI candidates) have significantly more psychosocial problems than their hearing peers (Barker et al., 2009; Hoffman, Quittner, & Cejas, 2015). However, there is also evidence that children with all degrees of hearing loss experience psychosocial problems (Bess, Dodd-Murphy, & Parker, 1998; Kouwenberg, 2013). Thus, for a sample of DHH children using HAs, Davis, Elfenbein, Schum, and Bentler (1986) reported no differences in emotional, behavioral, or social problems between children with mild, moderate, and severe losses, despite all children having significantly more social and behavioral problems than expected by comparison with norms.

Focusing on studies that have used the SDQ with participant samples composed predominantly of children with HAs, the majority have found significantly higher rates of psychosocial difficulties compared with hearing children and no significant effect of degree of hearing loss (Dammeyer, 2010; Fellinger et al., 2008; Hintermair, 2007; Stevenson et al., 2010). An Australian population study (Wake, Hughes, Collins, & Poulakis, 2004) looking at outcomes of DHH children with mild to profound loses (86% using HAs) found that, on average, the children had significantly more parent- and teacher-rated behavioral problems on the Child Behavior Checklist (CBCL), and lower adaptive skills and health-related QoL compared with the normative population. This pattern was found, despite the children having normal intellect, no additional disabilities, early amplification, and receiving ongoing intervention and support.

Overall, it appears that children with HAs who have mild to profound levels of hearing loss may all be at risk of poor psychosocial development. Some researchers have argued that partial or mild degrees of hearing loss may even affect self-concept more because being “so close to normal” makes acceptance of disability more difficult (Mykleburst, 1960, cited in Polat, 2003). Again, however, before drawing strong conclusions, the contribution of other potential mediating factors needs to be examined.

### Factors Influencing Outcomes in Children With HAs

Language ability, functional auditory behavior, child-related (i.e., additional disabilities, non-verbal cognitive ability), and family-related (i.e., home communication mode, maternal education, and SES) factors may contribute to psychosocial outcomes for all DHH children regardless of hearing device (Leigh et al., 2015; Polat, 2003; Theunissen et al., 2014c). Similar to children with CIs, earlier age of intervention in the form of HA fitting may also contribute to better psychosocial outcomes (Hind & Davis, 2000; Hoffman et al., 2015; Yoshinaga-Itano, Sedey, Coulter, & Mehl, 1998), although such an advantage has not consistently been found (Stevenson et al., 2011).

A factor that is relevant for children with HAs specifically and has been shown to influence language outcomes is the child’s degree of hearing loss. Studies looking at the relationship between severity of hearing loss and psychosocial development have been varied. Wake et al. (2004) found that, although severity of hearing loss was related to language ability, it was not significantly associated with psychosocial outcomes. It is worth noting, however, that while the differences were not significant, children with mild losses were rated as having the lowest QoL and the most behavior problems. In contrast, Fellinger et al. (2008) found that although the differences did not reach significance, children with severe levels of hearing loss had a higher incidence of externalizing problems (e.g., conduct and hyperactivity) on the SDQ compared with those with moderate or profound losses (including children with HAs and CIs). In their review of the evidence, Theunissen, Reiffe, Netten, et al. (2014b, 2014c) reported that most literature has reported no significant influence of degree of hearing loss on psychosocial outcomes.

### Summary of Evidence

In summary, the majority of previous literature has reported psychosocial difficulties in DHH children compared with hearing children. There are relatively few studies focusing on children with HAs compared with those with CIs. Although a number of studies have found that children with CIs perform at similar levels to hearing children on psychosocial measures (Anmyr et al., 2012; Huber & Kipman, 2011), this pattern has not been found consistently (Dammeyer, 2010; Huber, 2005; Peterson, 2004). A number of researchers have concluded that the psychosocial well-being of children with CIs lies somewhere between that of hearing children, and that of children with similar levels of hearing losses using HAs (Dammeyer, 2010; Theunissen et al., 2012; Theunissen et al., 2014a). In regard to factors that influence outcomes, there is little evidence that severity of hearing loss influences psychosocial development. In contrast, there is more substantial evidence that age at intervention, and child- and family-related factors influence outcomes for DHH children using CIs or HAs. However, many of these factors may only indirectly impact psychosocial outcomes through their influence on language and functional auditory behavior (Wong et al., 2016).

In regard to the published literature, it is noteworthy that few population studies have examined psychosocial development in pre-school age DHH children who have had access to early intervention. Previous studies have included children with a wide age range, despite the fact that age has been significantly associated with psychosocial outcomes in DHH children (Nicholas & Geers, 2003; Polat, 2003). Furthermore, outcome measures have been heterogeneous (although the SDQ is most consistently used), and prevalence rates may be inflated due to the use of different cut-off scores or sampling of participants from schools for the deaf. Finally, very few studies have looked at psychosocial functioning at a range of levels from basic social skills to peer relations and emotional or behavioral difficulties (Batten et al., 2014). Although these psychosocial factors are interrelated, they are theoretically separate constructs. Exploring psychosocial outcomes at these different levels is, therefore, important. Although a DHH child may have inferior social skills or few friends, it will not necessarily be reflected in poor mental health or QoL.

### Aims

To address the issues raised earlier, the aims of the current study were to as follows (a) examine the psychosocial outcomes of 5-year-old DHH children compared with normative data separately for those using CIs or conventional HAs, (b) compare the outcomes of children with CIs to those with similar levels of hearing losses using HAs, and (c) investigate the factors influencing psychosocial outcomes separately for children with CIs or HAs.

Based on the balance of evidence, it was hypothesized that (a) both children with CIs and HAs would show more psychosocial problems compared with normative data, (b) for children with similar levels of hearing loss, those using CIs would be rated as having fewer psychosocial problems than children with HAs, and (c) the same factors would influence psychosocial outcomes in children with CIs and HAs, with the influence of hearing loss mediated predominantly by variation in language and functional auditory behavior.

## Method

### Participants

The data presented here were collected as part of the Longitudinal Outcomes of Children with Hearing Impairment (LOCHI) study—a longitudinal study that has prospectively measured the language, psychosocial, and educational outcomes of a large cohort of Australian children with hearing loss. Detailed information about the LOCHI study has been presented previously by Ching, Leigh, and Dillon (2013). In brief, families with children born between May 2002 and August 2007 in the Australian states of New South Wales, Victoria, and Queensland who were identified with hearing loss and fitted with amplification before 3 years of age were invited to participate in the study. All children in this study had access to the same hearing service provider (Australian Hearing) before 3 years of age. Written parental consent was obtained for each participant, and ethics was approved for the study from the Australian Hearing human research ethics committee (EC00109).

Data on at least one psychosocial measure were available for 356 children enrolled in the LOCHI study when they were turning 5 years old (*M* = 61.6 months, *SD* = 1.9; range 58–73 months). There were 317 CDI forms and 333 SDQs completed by parents, with 294 parents completing both measures. Children who were no longer using hearing devices at 5 years (*n* = 3) were excluded from the current analyses. In total, the study cohort comprised of 194 males and 162 females. More children wore HAs (66.3%) than CIs (33.7%).

### Measures

#### Psychosocial measure: CDI

The CDI (Ireton, 1992) is a parent-rated standardized questionnaire designed to assess children’s development from 15 months to 6 years of age. Although the CDI has eight subscales, we focus on one CDI subscale that describes aspects of social skills development. The Social subscale of the CDI (40 items) measures aspects of personal and group interaction and social behaviors, including care and concern for others (e.g., “shows sympathy to other children”), initiative (e.g., “asks for help in doing things”), independence (e.g., “shows leadership among children his/her age”), and social interaction (e.g., “makes or builds things with other children”).

Published normative data (Ireton & Glascoe, 1995) were used to recalculate children’s individual results into developmental ages, which were then used to derive *Z* scores. *Z* scores were calculated by subtracting the child’s chronological age from developmental age and dividing this by 1 *SD* (i.e., 15% of the chronological age norms according to Ireton & Glascoe, 1995). There were 317 completed forms returned for the CDI. Missing data were most commonly due to the forms not being returned.

#### Psychosocial measure: SDQ

The SDQ (Goodman, 1997) is a 25-item screening measure designed to identify behavioral and emotional problems in children. The instrument consists of five subscales: conduct problems (e.g., fights with others), hyperactivity (e.g., restless or easily distracted), emotional symptoms (e.g., many worries, often unhappy), peer problems (e.g., picked on or bullied), and prosocial behavior (e.g., considerate of others feelings). Each subscale consists of five items rated on a 3-point response scale from 0 = *not true*, 1 = *somewhat true* to 2 = *certainly true*. Scores from each domain (excluding prosocial behavior) were summed to make a “total difficulties score.” Higher scores on the prosocial domain reflect strengths, whereas higher scores on the remaining subscales and total difficulties scores indicate more emotional and behavioral problems. *Z* scores were calculated from recent Australian normative data of children aged 5 years (Kremer et al., 2015). All difficulties scores were reversed so that higher *Z* scores reflected better psychosocial functioning. There were 333 completed forms returned for the SDQ. Missing data were most commonly due to the forms not being returned.

#### Language: Preschool Language Scale—fourth edition

The Preschool Language Scale—fourth edition (PLS-4; Zimmerman, Steiner, & Pond, 2002) is a standardized language test used to identify language disorders between birth and 6 years 11 months. The test contains two subscales of Expressive Communication (EC) and Auditory Comprehension (AC), which are combined to derive a “total language score.” The EC subscale items for preschool age include naming of common objects, using concepts to describe objects, defining words, and using grammatical constructions. The AC subscale includes items that assess comprehension of vocabulary, concepts, complex sentences, and drawing inferences. Standard scores and age-equivalent scores were calculated using normative data. There were 25 children in the study who were not able to complete the PLS-4 due to various reasons, which included the following: being from a non-English speaking background, not wearing their CI or HA on the day of testing, not being available for testing, compliance issues, or being unable to cope with the level of testing. An additional 21 children required the PLS-4 to be administered using simultaneous communication methods (i.e., a combination of sign and oral) making calculation of standard scores inappropriate for this small subset of children.

#### Functional auditory behavior: The Parents’ Evaluation of Aural/Oral Performance of Children

The Parents’ Evaluation of Aural/Oral Performance of Children (PEACH; Ching & Hill, 2007) is a measure of functional auditory and communicative performance in everyday life as judged by caregivers. The test contains 13 questions, 2 of which address the child’s use of sensory devices. The remaining 11 questions solicit information about the child’s ability to listen and communicate in quiet and in noise, to use the telephone, and to respond to environmental sounds in everyday situations. An overall functional performance score was calculated using the summed ratings provided by caregivers in response to the 11 questions. Higher scores reflect better listening outcomes for all sounds. *Z* scores were derived from published normative data on children with normal hearing (Ching & Hill, 2007). There were 299 PEACH forms completed and returned. Missing data were mostly due to the forms not being returned.

#### Non-verbal cognitive ability: Wechsler Non-Verbal Scale of Ability

Non-verbal cognitive ability was assessed using the Wechsler Non-Verbal Scale of Ability (WNV; Wechsler, Naglieri, & Petermann, 2006). The WNV is a standardized assessment specifically devised for linguistically diverse populations, including people with hearing loss. The assessment comprises four subtests—matrices, coding, object assembly, and recognition (for children ages 4years–7 years and 11 months), which combine to provide a full-scale IQ score. WNV scores were obtained from 288 children. There were 68 children who were unable to complete the WNV test: 22 were unable to cope with the demands of the test, 38 were unavailable for testing, and 8 failed to complete for “other” reasons.

### Procedure

Each child’s caregiver completed the earlier questionnaires and a custom-designed demographic questionnaire. The demographic questionnaire included questions concerning the child’s diagnosed disabilities in addition to hearing loss, communication mode used at home and in early intervention (spoken only, sign only, or a combination), location of residence (residential postcode), and the caregivers’ own educational experience. SES was measured using the Index of Relative Socio-economic Advantage and Disadvantage (Australian Bureau of Statistics, 2006), which is expressed as a decile from 1 to 10 with higher scores indicating greater advantage.

Data regarding children’s age at first HA fitting, degree of hearing loss (i.e., better ear 4 frequency average, 4FA), type of hearing device, and age at CI switch-on, were provided by Australian Hearing (the Australian Government agency which provides audiological services for all Australian children who are residents or citizens). The child’s most recent available audiogram was used (i.e., within 6 months of the child completing the LOCHI language or cognitive assessment and parents completing questionnaires). The PLS-4 was administered as part of the larger LOCHI study when children were between 56 and 70 months (Mean age = 61.64, *SD* = 1.87). A speech pathologist administered the test at the child’s home or school. Non-verbal cognitive ability was also assessed at this time by a psychologist using the WNV.

### Analyses

All statistical analyses were conducted using SPSS Statistics Package 21 (IBM Corp, 2012). With respect to device, children were grouped into those with HAs or CIs. A single child who used only sign language to communicate was grouped with children who used a combination of speech and sign communication for analysis purposes. All test scores for the CDI (Ireton & Glascoe, 1995) and SDQ (Kremer et al., 2015) were converted to *Z* scores to allow for direct comparison between DHH children and normative data. To investigate for any group difference in psychosocial scores according to hearing device, one-way analyses of variance (ANOVAs) were conducted to compare children with CIs or HAs who had similar levels of hearing loss (i.e., ≥60dBHL). Non-parametric Chi square analyses were used to examine any demographic differences between groups of children with HAs or CIs.

To reduce the effect of measurement error and other random variations in individual test scores across the SDQ and CDI, the two scales were combined to make an aggregate “global psychosocial score” for the purpose of identifying the factors mediating psychosocial performance. This approach was supported by a factor analysis which indicated one underlying factor that accounted for 62% of the variance. The global score was calculated by averaging the *Z* scores from SDQ total difficulties score, SDQ prosocial score, and CDI social skills score. In total, there were 294 children who had completed both CDI and SDQ scales to make a global score.

Spearman Rho correlations were used to examine associations between child, family, audiological factors, language, functional auditory behavior, and the global psychosocial scores for children with HAs and CIs. To examine the unique associations between specific factors and psychosocial outcomes while controlling for other factors, hierarchical multiple regression analyses were run separately for children wearing HAs versus CIs, with the global psychosocial score as the dependent variable. However, there were only 67 children with CIs and 138 children with HAs who had complete data for all predictor variables.

Most common missing values were for direct child assessments of PLS-4 (*n* = 55) and WNV (*n* = 65), as well as PEACH forms (*n* = 58). More children with CIs had missing PLS-4 scores compared with those with HAs (*χ*^2 ^= 10.53, *p* = .001), but there was no difference between groups for WNV or PEACH. Little MCAR’s test was significant for the predictor variables which indicated the data were not “missing completely at random.” Some of the missing data in this cohort may be related to other known characteristics of the participants (e.g., additional disabilities, communication mode, etc.). Accordingly, significantly more children who used combined communication mode (*χ*^2 ^= 69.18, *p* < .001) and had additional disabilities (*χ*^2 ^= 9.16, *p* = .002) had missing PLS-4 scores. Missing WNV scores were also significantly more common in children with additional disabilities (*χ*^2 ^= 5.88, *p* = .015), but there was no difference in missing scores between children who used spoken or combined communication mode. Children from English-speaking and Non-English speaking backgrounds (NESB) did not differ with respect to completion of either PLS-4 or WNV. Due to these inconsistent results, as well as the wide range of variables collected from parents and children, it seemed reasonable to assume that most data were “missing at random.”

As removing cases with incomplete data introduces bias and a drop in statistical power, a multiple imputation technique was used to handle missing data for the regression analyses. First, attempts were made to manually impute missing scores for children who were unable to cope with testing. For missing data on WNV, basal scores (i.e., scaled score of 30) were assigned to three children who were unable to complete the tests due to severe intellectual disabilities. For the PLS-4, a basal score was assigned to one child who was unable to cope with the test demands, and who received a basal score in the CDI language subscale as rated by parents. A multiple imputations method (with 10 imputations) was used to handle the remaining missing data for the predictor variables. The variables used for imputing scores included gender, presence of additional disabilities, non-verbal cognitive ability, device, age of intervention, severity of hearing loss, maternal education, SES, PLS-4 language score, PEACH, CDI social skills, and SDQ prosocial and total scores. The outcome psychosocial variables themselves were not imputed to avoid introducing unnecessary noise to the estimates (Von Hippel, 2007).

Regression models were run in two steps to investigate the effect of language and functional auditory behavior on psychosocial outcomes after controlling for the effects of age at intervention, child- and family-related demographics. For children with HAs, the predictors in Model 1 included age at first HA fit, degree of hearing loss (better ear 4FA), non-verbal cognitive ability, presence of additional disabilities, maternal education, and communication mode. For children with CIs, predictors in Model 1 included age at CI switch on, non-verbal cognitive ability, presence of additional disabilities, maternal education, and communication mode. For both groups, Model 2 included the child’s language (PLS-4 total) and functional auditory behavior (PEACH) scores in addition to all variables in Model 1. The pooled analyses from 10 imputations were used and a significance value of *p* < .05 was used for all analyses.

## Results

### Demographics

Table 1 presents the demographic information relating to children in the HA and CI groups and their caregivers. Significant differences between the HA and CI groups are evident for communication mode (*χ*^2 ^= 14.19, *p* < .001) where a significantly higher proportion of children with CIs used a combination of sign and spoken communication mode (32.2%) compared with children with HAs (15%). In addition, significantly more children with HAs had parents with hearing loss (*χ*^2 ^= 10.01, *p* = .002).

**Table 1.** Demographic Information of Children With HAs and CIs.

	HA (*n* = 236)	CI (*n* = 120)	p^a^ (HA vs. CI)
Gender			
Male	137 (58.1%)	57 (47.5%)	ns
Female	99 (41.9%)	63 (52.5%)	
Age at assessment (CDI)			
Mean months (*SD*)	61.51 (1.93)	61.85 (1.85)	ns
Range	58–73	59–67	
Additional Disabilities			
No	145 (62.5%)	76 (63.3%)	ns
Yes	87 (37.5%)	39 (32.5%)	
Missing	4 (1.7%)	5 (4.2%)	
Communication mode			
Speech only	199 (84.3%)	80 (66.7%)	*P* < .001
Speech and sign	35 (14.8%)	37 (30.8%)	
Sign only	0	1 (1.7%)	
Missing	2 (0.8%)	2 (1.7%)	
Severity of hearing loss (4FA in the better ear)			
Mild (≤40 dB HL)	60 (25.4%)	0	*P* < .001
Moderate (41–60 dB HL)	120 (50.8%)	2 (1.7%)	
Severe (61–80 dB HL)	48 (20.3%)	9 (7.5%)	
Profound (>80 dB HL)	8 (3.4%)	109 (90.8%)	
Age at HA fit			
Mean months (*SD*)	10.68 (10.37)	6.44 (7.17)	
Range	1–35	1–34	
Age at CI switch on			
Mean months (*SD*)	N/A	16.27 (8.17)	
Range		4–59	
Maternal education			
High school	68 (28.8%)	38 (31.7%)	ns
Diploma	60 (25.4%)	28 (23.3%)	
University	96 (40.7%)	45 (37.5%)	
Missing	12 (5.1%)	9 (7.5%)	
Socio-economic status (IRSAD Decile)			
Mean (*SD*)	7.02 (2.56)	6.98 (2.63)	ns
Range	1–10	1–10	
Native language of parents			
English	117 (75%)	84 (70%)	ns
NESB	42 (17.8%)	21 (17.5%)	
Missing	17 (7.2%)	15 (12.5%)	
Parental hearing status			
No HL	190 (80.5%)	111 (92.5%)	*p* = .002
HL in one parent	43 (18.2%)	4 (3.3%)	
HL in both parents	3 (1.3%)	4 (3.3%)	
Missing		1 (0.8%)	

*Note.* HA = hearing aid; CI = cochlear implant; 4FA = 4 frequency average; dB HL = decibel hearing level, NESB = non-English speaking background. ^a^Chi square or ANOVA test (ns *p* > .05).

### Psychosocial Functioning of Children With CIs Compared With Normative Data

Table 2 shows the means and *SD*s, expressed in terms of *Z* scores derived from published normative data (Ireton & Glascoe, 1995; Kremer et al., 2015), for each psychosocial measure, language ability, functional auditory behavior, and non-verbal cognitive ability. Results are presented separately for three groups of children: all HA users, HA users with a severe to profound hearing loss (≥60 dBHL), and all CI users. On average, SDQ subscale scores for children with CIs were between .02 and .40 *SD*s below the norm. Individual subscale ratings indicate the fewest problems with emotional difficulties, and the most with conduct. In contrast, the mean CDI social skills score fell 1.40 *SD*s below the norm. There were no significant gender differences on SDQ or CDI subscales with the exception of peer problems: boys were rated as having significantly more peer problems (*Z* = −.57) than girls (*Z* = −.06). Overall, the global psychosocial score was .60 *SD*s below the norm.

**Table 2.** Mean *Z* scores (*SD*s) of Psychosocial, Language, and Functional Communication Scores for Children With HAs and CIs.

	HA-all HL	HA-severe	CI	p^a^ (HA-all vs. CI)	p^a^ (HA-severe vs. CI)
*SDQ*	*n = 223*	n = 54	*n = 110*		
Emotion	−.031 (.985)	.037 (1.02)	−.019 (1.07)	*n.s*	*n.s*
Conduct	−.607 (1.20)	−.685 (1.40)	−.409 (1.10)	*n.s*	*p = .039*
Hyperactivity	−.436 (1.20)	−.809 (1.44)	−.235 (.991)	*n.s*	*p = .02*
Peer problems	−.270 (1.22)	−.384 (1.12)	−.297 (1.22)	*n.s*	*n.s*
Prosocial	−.272 (1.17)	−.232 (1.30)	−.216 (1.17)	*n.s*	*n.s*
Total	−.513 (1.08)	−.697 (1.14)	−.374 (1.01)	*n.s*	*n.s*
n (%) ≥2*SD*s norm	29 (13%)	8 (14.8%)	10 (9.1%)	*n.s*	*n.s*
*CDI*	n = 210	n = 49	*n = 107*		
Social skills	−1.45 (1.98)	−1.69 (2.13)	−1.37 (1.90)	*n.s*	*n.s*
n (%) ≥2*SD*s norm	95 (45.2%)	28 (57.1%)	44 (41.1%)	*n.s*	*n.s*
*Global Psychosocial Score*	n = 197	n = 47	n = 97		
	−.692 (1.04)	−.805 (1.13)	−.590 (1.06)	*n.s*	*n.s*
*Language*	*n = 212*	n = 44	*n = 93*		
PLS-4 AC	−.978 (1.29)	−1.45 (1.25)	−1.14 (1.38)	*n.s*	*n.s*
PLS-4 EC	−.964 (1.22)	−1.45 (1.21)	−1.38 (1.48)	*p = .014*	*n.s*
Total	−1.01 (1.31)	−1.55 (1.25)	−1.35 (1.50)	*p = .049*	*n.s*
*Func Aud Beh*	*n = 192*	n = 46	*n = 106*		
PEACH	−.722 (.884)	−.912 (.975)	−.621 (.923)	*n.s*	*n.s*
*Non-verbal cog*	n = 196	n = 42	n = 95		
WNV	.168 (1.14)	−.027 (1.18)	.024 (1.12)	*n.s*	*n.s*

*Note*. SDQ = Strengths and Difficulties Questionnaire; CDI = Child Development Inventory; PLS-4 = Preschool Language Scale-4; AC = auditory comprehension; EC = expressive communication; Func Aud Beh = Functional auditory behavior; PEACH = Parent Evaluation of Aural/Oral Performance of Children; WNV = Wechsler Non-Verbal Scale of Ability. *p*^a^ ANOVA or Chi square tests; ns = not significant (*p* > .05); ≤2 *SD*s norm = proportion falling more than 2 *SD* below same-age peers. Due to missing data for some variables, scores are based on different numbers of participants as specified.

Table 2 also shows the proportion of children falling 2 *SD*s or below age norms. These low scores are considered to reflect clinically significant emotional or behavioral difficulties (i.e., ≤ 2nd percentile). Nine percent of children with CIs fell more than 2 *SD*s below the norm on the SDQ total score and 41.1% fell below expected age level on the CDI social skills (compared with 2.5%–3% in the general population). Overall, children with CIs fell 1.35 *SD*s below the norm on expressive and receptive language skills. PEACH scores fell approximately .60 *SD*s lower than hearing children. Non-verbal cognitive ability was on average, close to the norm (*Z* = .024).

### Psychosocial Functioning of Children With HAs Compared With Normative Data

Similar to children with CIs, children with HAs scored on average between .03 to .60 *SD*s below norms on SDQ subscales, whereas the mean score on the CDI social skills scale was 1.45 *SD*s below norms. On the SDQ, individual subscale ratings indicate the fewest problems with emotional difficulties, and the most problems with conduct and hyperactivity. There were no significant gender differences on any psychosocial measure. Overall, the mean global psychosocial score was .69 *SD*s below the norm. As shown in Table 2, the proportion of children falling ≥2 *SD*s below the norm was 13% on the SDQ total score, and 45% on CDI social skills. In regard to language and functional auditory behavior, children had language scores that fell close to 1 *SD* below normative data on expressive and receptive language skills overall. Functional auditory behavior (PEACH) scores fell approximately .70 *SD*s below those of hearing children. On average, non-verbal cognitive ability fell within the normal range.

### Differences Between CIs and HAs for Children With Similar Levels of Hearing Loss

Differences between CI and HA groups (using ANOVA or *χ*^2^ analyses) are shown in Table 2. When comparing children with CIs to those in the HA-severe group (i.e., ≥60 dBHL), analyses of variance revealed significant differences on the SDQ behavioral subscales of hyperactivity, *F* (1, 161) = 4.31, *p* = .039, and conduct difficulties, *F* (1, 161) = 5.48, *p* = .02, with more difficulties evident for children using HAs. By contrast, these groups did not differ significantly on CDI social skills, *F* (1, 152) = 1.01, *p* = .317, emotional difficulties, *F* (1, 161) = .110, *p* = .740, peer problems, *F* (1, 161) = .386, *p* = .535, or prosocial behavior, *F*(1, 161) = .037, *p* = .848, and no significant group difference was seen in the proportion of children who fell ≥2 *SD*s below norms on either the SDQ or the CDI. When comparing the group of CI users to the group of all HA users (HA-all), there were no significant differences on any psychosocial measure.

### Factors Influencing Psychosocial Outcomes for Children With CIs

Table 3 presents the correlations between demographic variables, language, functional auditory behavior, and global psychosocial outcomes for children with CIs and HAs separately. In the group of children with CIs, higher global psychosocial outcomes were significantly correlated with an absence of additional disabilities, use of spoken only communication, better language, and better functional auditory behavior. By contrast, there was no significant association with non-verbal cognitive ability (WNV), age at CI switch on, or maternal education.

**Table 3.** Spearman Rho Correlations Between Child, Audiological, Family Factors, Language, Functional Auditory Behavior and Psychosocial Scores for Children With CIs and HAs.

CI children	Disabil N:Y	Age CI switch on	Comm Mode	Mat Ed	Language	Func Aud Beh	CDI social skills	SDQ total	Global psychosocial	
WNV	−.359**	−0.033	−.247*	.198*	.487**	0.111	.256*	.124	0.178	
Disabil		−0.054	.446**	0.134	−.393**	−0.182	−.306**	−.328**	−.357**	
CI switch on			0.158	−.236*	−.293**	−0.087	−.037	−.020	0.038	
Comm Mode				0.008	−.307**	−.240*	−.233*	−.220*	−.230*	
Mat ed					.245*	0.155	−.006	.230*	0.115	
Language						.347**	.397**	.305**	.361**	
Func Aud Beh							.413**	.407**	.463**	
CDI social								.367**	.880**	
SDQ Total									.656**	
HA children	Disabil N:Y	Age First HA fit	Comm Mode	4FA	Mat Ed	Language	Func Aud Beh	CDI Social Skills	SDQ Total	Global Psychosocial
WNV	−.394**	−0.059	−.144*	−0.102	.248**	.534**	.286**	.511**	.399**	.483**
Disabil		0.107	.215**	0.049	−0.105	−.347**	−.304**	−.384**	−.235**	−.340**
First HA fit			−0.047	−.194**	−0.076	−0.130	−.210**	−123	−**.108**	−0.123
Comm Mode				.188**	−0.029	−.194**	−0.126	−.247**	−.162*	−.172*
4FA					.188**	−0.013	−.258**	−.108	−**.119**	−.154*
Mat ed						.264**	.296**	.202**	.291**	.285**
Language							.346**	.570**	.439**	.546**
Func Aud Beh								.384**	260**	.383**
CDI social									.459**	.870**
SDQ Total										.736**

*Note*. Disabilities (no [reference], yes); 4FA = better ear 4 frequency average; Comm Mode = communication mode (spoken only [reference], combined); Mat ed = maternal education; Func Aud Beh = functional auditory behavior (PEACH).

* *p *< .05. **p < .001.

Multiple regression analyses were conducted to identify variables accounting for unique variance in global psychosocial functioning. When all child, family-, and intervention-related factors were included in Model 1, the only significant demographic variable was the presence of additional disabilities (*B* = −.707, 95%CI [−1.2, −.2], *p* = .006, see Table 4). Children with additional disabilities scored on average .7 *SD*s lower on global psychosocial functioning than children without additional disabilities after controlling for other demographic factors. In contrast, non-verbal cognitive ability, age at CI switch on, communication mode, and maternal education did not account for significant unique variance in outcomes. Together, all demographic variables explained 14.3% of the variance in global psychosocial function. The addition of language and functional auditory behavior in Model 2, substantially and significantly increased the proportion of variance explained by 17.3% (to a total *R*^2 ^= .316). However, only functional auditory behavior accounted for significant unique variance in global psychosocial functioning (*B* = .479, 95%CI [.251, .706], *p* < .001), whereas language score did not. Presence of additional disabilities remained a significant factor after controlling for language and functional auditory abilities. To examine the independent variance explained by functional auditory behavior, a simple linear regression was conducted with PEACH score as the only predictor: This single variable uniquely explained 22% of the variance in global psychosocial functioning (*B* = .590, *p* < .001).

**Table 4.** Regression Analyses Predicting Global Psychosocial Functioning for Children With HAs and CIs.

	Model 1			Model 2		
	B	95%CI		B	95%CI	
CI children						
WNV SS	.003	−.017	.023	.000	−.018	.019
Disabilities	−.707**	−1.214	−.200	−.560*	−1.058	−.061
Age switch on	.007	−.009	.022	.006	−.009	.020
Comm mode	−.394	−.866	.079	−.199	−.639	.241
Maternal Ed	.197	−.056	.450	.062	−.173	.297
Language				.073	−.115	.261
Func Aud Beh				.479**	.251	.706
Adjusted r2	.143			.316		
HA children						
WNV SS	.026**	.017	.034	.018**	.008	.027
Disabilities	−.312*	−.600	−.025	−.140	−.419	.139
Age first fit	−.002	−.010	.006	.004	−.003	.012
4FA	−.007	−.018	.004	−.003	−.014	.007
Comm Mode	−.257	−.614	.100	−.119	−.462	.223
Maternal Ed	.215**	.060	.370	.063	−.092	.217
Language				.230**	.107	.353
Func Aud Beh				.233**	.072	.394
Adjusted *R*^2^	.332			.417		

*Note.* SS = scaled score; Disabilities (no [reference], yes); 4FA = better ear 4 frequency average; Comm Mode = communication mode (spoken only [reference], combined); Func Aud Beh = functional auditory behavior (PEACH).

* *p* < .05. **p < .001.

### Factors Influencing Psychosocial Outcomes for Children With HAs

For HA users, global psychosocial outcomes were significantly correlated with nearly all demographic and audiological variables including non-verbal cognitive ability, presence of additional disabilities, communication mode, degree of hearing loss, and maternal education (See Table 3). Only age of first HA fit was not significantly correlated. Results from multiple regression analyses summarized in Table 4 show that three demographic variables were significant associated with better global psychosocial function after controlling for all other variables. These associated variables were higher non-verbal cognitive ability (*B* = .026, 95%CI [.017, .034], *p* < .001), absence of additional disabilities (*B* = −.312, 95%CI [−.6, −.025], *p* = .033) and higher levels of maternal education (*B* = .25, 95%CI [.06, .37], *p* = .006). There was no significant effect of age at first fit, 4FA, or communication mode. All variables in Model 1 accounted for 33.2% of the variance in global psychosocial function. The addition of language and functional auditory behavior in the second model significantly increased the proportion of variance explained by 8.1% (total *R*^2 ^= .417). Both language (*B* = .230, 95%CI [.107, .353], *p* < .001) and functional auditory behavior scores (*B* = .233, 95%CI [.072, .394], *p* < .001) were significantly associated with global psychosocial function, although functional auditory behavior had a slightly stronger effect size (i.e., a larger regression coefficient and 95%CI). In the final model, only non-verbal cognitive ability remained a significant demographic mediator of global psychosocial function, whereas additional disabilities and maternal education were no longer significant. To examine the independent variance explained by language and functional auditory behavior alone, we conducted simple linear regressions predicting global psychosocial scores. Functional auditory behavior uniquely explained 13.5% of the variance in global scores (*B* = .475, *p* < .001), whereas language ability uniquely explained 26.9% of the variance (*B* = .423, *p* < .001).

## Discussion

The aims of this study were to (a) examine the psychosocial functioning of 5-year-old DHH children and compare their outcomes to normative data separately for children with CIs and children with HAs, (b) compare the outcomes of children with similar levels of hearing loss who use CIs or HAs, and (c) investigate the potential factors influencing outcomes separately for children with CIs and children with HAs. Our first hypothesis, that both children with CIs and children with HAs would show more psychosocial problems compared with normative data were only somewhat supported. On average, DHH children in this cohort with CIs or HAs scored within 1 *SD* of the norm in regard to emotional or behavioral problems on the SDQ; however, they showed delayed social skill development on the CDI, with ratings on average falling 1.4 *SD*s below the typical norm. The second hypothesis was also partially supported for children with severe to profound hearing losses, where children with HAs showed significantly more parent-rated behavioral problems (hyperactivity and conduct) than children with CIs, but no differences were found in social skills or emotional difficulties. Against our final hypothesis, some differences between the two hearing device groups were apparent in regard to demographic and audiological factors associated with global psychosocial outcomes. For children with HAs, significant correlates of better global psychosocial functioning included higher non-verbal cognitive ability, language abilities (PLS-4), and functional auditory behavior (PEACH), after controlling for all other variables. For children with CIs, the presence of additional disabilities and functional auditory behavior significantly mediated psychosocial outcomes, whereas language scores did not. Only functional auditory behavior was a strong and consistent mediator of global psychosocial outcomes for both children with CIs and HAs.

### Psychosocial Functioning of Children With CIs

The current results show that, on average, DHH children with CIs scored within 1*SD* of typically developing children on the SDQ and the global psychosocial score. This result is in line with previous studies (Anmyr et al., 2012; Huber & Kipman, 2011; Percy-Smith, Cayé-Thomasen, et al., 2008), showing that children with CIs are comparable to their hearing peers on psychosocial measures. Improvements in early identification, intervention and education efforts for the current generation of DHH children might partly explain these findings. On the SDQ total difficulties score, children scored .37 *SD*s lower than norms. This outcome is consistent with the recent review by Stevenson et al. (2015), which found that DHH children (with HAs and CIs) fell .23 *SD*s below hearing children across included SDQ studies. On the other hand, borderline delays were reported on the CDI social skills subscale, on which children with CIs fell an average of 1.37 *SD*s below age-norms. The difference in outcomes between the SDQ and CDI is likely due to their tapping different aspects or levels of psychosocial functioning, as well as the age of the current sample. The social skills subscale of the CDI was designed specifically for children under 6 years of age, and covers more specific early interactive skills (e.g., plays physical games with other children, plays “pretend” games with other children) and many items that rely on verbal or communicative skills (e.g., “greets people with ‘hi’”; “says ‘I can’t’, ‘I don’t know,’ or ‘You do it’”). By contrast, the SDQ covers domains related to more clinical problems, such as emotional and behavioral disorders, and is targeted at school-aged children aged 4 and 17 years. The CDI also has a much higher number of items, and a bimodal response choice compared with the SDQ. These differences may partly explain why children were rated as having more difficulties on the CDI than the SDQ.

Although average scores on the SDQ fell within the range of typically developing children, 9% of children with CIs had scores more than 2*SD*s below the mean, indicating problems of potential clinical significance. This proportion is higher than would be expected in the general population (2.5%). Indeed, it is more similar to findings from previous studies that have used the proportion of children who fall below clinical “cut-off” scores to identify emotional or behavioral problems rather than using average scores or effect sizes (Fellinger et al., 2008; Hintermair, 2007). However, Remine and Brown (2010) cautioned that some previous studies identified children with both borderline and clinical scores (i.e., bottom 20%) as having mental health problems, which may have artificially inflated prevalence rates. Given the wide variability evident in psychosocial outcomes within DHH children, it is important to consider the heterogeneity within this group.

The language outcomes of the current cohort of children with CIs, fell on average 1.3 *SD*s below the general population, in line with their language outcomes at 3 years of age (Ching, Dillon, et al., 2013). This result indicates that, on average, children with CIs still lag behind their hearing peers in language development, despite early identification. More in-depth investigation of the language outcomes of the LOCHI children at 5 years of age is presented in Cupples et al. (in press). In the context of the current findings, however, delayed language scores may have an immediate and direct impact on early social skills, whereas emotional and behavioral problems may arise later as the children grow older and enter formal schooling. In addition, as the current cohort is young and outcomes are dependent on parent ratings, it is not clear whether ratings may differ when the children are older and able to provide self-report. Anmyr et al. (2012) found that DHH adolescents tended to rate themselves as having more problems on the SDQ compared with parents and teachers. Overall, however, ratings were still within the range of typically developing children. The prospective nature of the LOCHI study will allow us to determine whether changes in psychosocial functioning and mediating factors occur as the demands of social and school dynamics become more complex.

### Psychosocial Functioning of Children With HAs

On average, children with HAs (and all levels of hearing loss) performed similarly to children with CIs. Their scores were within 1*SD* of the norm on the SDQ (.5 *SD*s below norms for total difficulties), but 1.45 *SD*s below norms on the CDI social skills scale. The mean global psychosocial score for the group of all children with HAs fell within the range of typically hearing children (.69 *SD*s below norms). These results indicate that, regardless of hearing device, 5-year-old DHH children do not have significantly more emotional or behavioral problems on average than their hearing age-matched peers, although they are at increased risk of delayed social skill development. This finding is consistent with a number of previous studies that have examined DHH children with HAs and CIs and found them to be comparable to the hearing population in regard to internalizing and externalizing problems, but to show evidence of significantly more peer problems (Huber et al., 2015; Remine & Brown, 2010; Stevenson et al., 2015). It has been posited that delays in strategic and pragmatic language and communicative skills might contribute to peer problems (Huber et al., 2015; Stevenson et al., 2015), but regardless, peer and social skill problems in DHH children do not necessarily equate to increased mental health problems. The longitudinal nature of the LOCHI study will, however, enable a better understanding of whether poor social skills at 5 years of age increase the risk for later psychosocial problems.

### Differences Between Children With HAs and CIs With Similar Levels of Hearing Loss

In line with our second hypothesis, children with HAs who had severe to profound losses showed evidence of significantly more psychosocial problems than children with CIs, but only in the areas of hyperactivity and conduct. As such, the findings are partly consistent with those reported in Theunissen et al. (2014a), who found increased rates of behavioral problems (including aggression, psychopathy and conduct disorder as measured on the Child Symptom Inventory) for children with HAs (who had moderate to profound losses) but not for children with CIs. Behavioral difficulties in typically developing children as well as children with other disorders such as ADHD and conduct disorder are typically thought to manifest from poor communication and self-regulatory abilities (Barkley, 1997; Clark, Prior, & Kinsella, 2002). The benefits of cochlear implantation may include enhanced speech perception skills, communication, and a greater number of social opportunities (Bat-Chava et al., 2005), thus in turn reducing behavioral problems. This interpretation receives some limited support from the current finding that children with HAs in the severe to profound range scored lower on functional auditory behavior (PEACH scores) and language scores compared with those in the CI group, although this difference did not reach significance.

In contrast to Theunissen et al. (2012) who investigated internalizing problems in DHH children, we did not find any differences in regard to emotional problems. However, compared with the current study, Theuniessen et al. included an older sample (mean age = 11 years, range 9–16) and used a more comprehensive screen of emotional disorders (Child Symptom Inventory). Again, emotional difficulties may increase with age and ability to self-report internalized issues (Hymel, Rubin, Rowden, & LeMare, 1990; Roza, Hofstra, van der Ende, & Verhulst, 2003). It will be important to monitor these aspects in the LOCHI sample because early behavioral problems have been linked to later mental health disorders (Caspi, Moffitt, Newman, & Silva, 1996).

### Factors Influencing Psychosocial Outcomes for Children With CIs

In the present investigation, factors influencing outcomes were examined separately in children with HAs and children with CIs. Some differences were found regarding the child and family-related variables that were associated with outcomes. For children with CIs, only the presence of additional disabilities was associated with lower global psychosocial function, whereas non-verbal cognitive ability, age of CI switch on, communication mode, and maternal education were not. For this group, all child-, family-, and intervention-related factors together explained 14.3% of the variance in global psychosocial functioning. This proportion of explained variance was smaller than for the group of HA users, perhaps because of the smaller sample size and the non-significant correlations between psychosocial outcomes and WNV, age at intervention and maternal education. Previous studies that found an effect of age at implantation in children with CIs were carried out in older children who had been implanted later (e.g., mean age 4 years) (Bat-Chava et al., 2005; Percy-Smith, Hedegaard Jensen, et al., 2008; Theunissen et al., 2012). In contrast the current CI cohort had their first CI switched on at 16 months of age, on average, and their first HA fitted at 6 months. The overall earlier age at intervention compared with previous studies may have reduced the impact of this variable on psychosocial outcomes. Although communication mode was not significantly associated with outcomes in the regression models, the direction was such that children with spoken only communication scored better than those using a combined mode. It is important to acknowledge, however, that children with CIs who use a combined communication mode may do so for other reasons such as the presence of additional disabilities or later implantation. Thus, once these factors are controlled, communication mode does not influence psychosocial outcomes.

Previous research has found that children with CIs and additional disabilities are more likely to have significant delays in expressive and receptive language (Meinzen-Derr, Wiley, Grether, & Choo, 2010), which may in turn, impact on social development. However, the current results show that the presence of additional disabilities remained a significant mediator of psychosocial outcomes after controlling for language in the final regression model. Interestingly, presence of additional disabilities was only significant for children with CIs but not HAs. Our future research will investigate in greater detail the psychosocial outcomes achieved by DHH children with additional disabilities to see whether there are specific additional disabilities or comorbidities that lead to higher risk of psychosocial dysfunction.

When language and functional auditory behavior scores were added into the regression model for children with CIs, the proportion of explained variance in global psychosocial outcomes increased by 17.3%, to a total of 31.6%. However, only functional auditory behavior (and not language) accounted for significant unique variance in global psychosocial outcomes for children with CIs. A simple linear regression including functional auditory behavior as the only predictor accounted for 22% of the variance in global psychosocial outcomes. The finding that language scores did not account for significant unique variance in psychosocial outcomes for this group may be attributed to the fact that more children with CIs had imputed PLS-4 standard scores, thus increasing variability. Nevertheless, the current findings are consistent with Huber & Kipman’s (2011) study, which found that CI users with good auditory performance had fewer problems on the SDQ. Ketelaar, Wiefferink, Frijns, Broekhof, & Rieffe (2015) also found that general language skills were not related to emotional or social functioning in children with CIs, although their emotional vocabulary did show an association. Ketelaar et al. (2015) concluded that it is not just the ability to understand and produce language that is critical for good social functioning, but rather the ability to communicate and use emotional language.

Children with CIs have been reported to show “social deafness” (Punch & Hyde, 2011b), which means that they have more difficulties in challenging listening environments, such as talking in a group of people, or in a classroom or playground situation, compared with one-on-one interactions. As social situations generally occur in informal settings where there may be background noise and distractions, children with hearing loss may have difficulty entering or participating in conversations, or detecting subtle cues in conversation. These difficulties can, in turn, lead to other children viewing their behavior as abnormal or negative, and ultimately to social isolation (Sorkin et al., 2015). The current findings indicate that the PEACH may be a useful screening tool for these children, as it is easy to administer and has the benefit of being applicable for even very young children, or children who cannot complete standardized testing due to additional disabilities or NESB.

### Factors Influencing Psychosocial Outcomes for Children With HAs

For children with HAs, non-verbal cognitive ability, presence of additional disabilities and maternal education were significantly associated with global psychosocial outcomes in the first regression model. All child, family, and intervention factors explained 33.3% of the variance in global psychosocial outcomes. However, the impact of most of those demographic variables on psychosocial functioning appeared to be indirect and driven through their influence on language and functional auditory behavior. When language and functional auditory behavior scores were entered into the final model, additional disabilities and level of maternal education were no longer significantly associated with outcomes for children with HAs.

Only non-verbal cognitive ability remained a significant and direct mediator of global psychosocial functioning. This result might indicate that higher intellectual ability is a protective factor for psychosocial development, possibly because children with higher cognitive ability are more adaptive in different situations (Kushalnagar et al., 2007). The finding that non-verbal cognitive ability is positively associated with social–emotional development in DHH children is consistent with a number of previous studies (Theunissen et al., 2014a; Van Eldik, 2005). However, previous studies have combined children with HAs and children with CIs. In the current study, non-verbal cognitive ability was significantly associated with psychosocial outcomes only for children with HAs and not for children with CIs, although variability was similar between groups. Potential reasons include differences in sample size and higher parental expectations for children with CIs and normal IQ to achieve outcomes similar to their hearing peers (Spahn, Richter, Burger, Löhle, & Wirsching, 2003).

Maternal education was significantly associated with global psychosocial outcomes for children with HAs in the first regression model. This result is consistent with the literature on hearing children, which indicates that low maternal education is a risk factor for child behavior problems (Fox, Platz, & Bentley, 1995; Gortmaker, Walker, Weitzman, & Sobol, 1990). However, this variable was no longer significant after accounting for children’s language ability and functional auditory performance, suggesting that maternal education influences psychosocial outcomes only indirectly. This result is in line with evidence from the general developmental literature, showing that maternal education and SES influence child language outcomes through maternal speech input that was higher in quantity, lexical richness, and sentence complexity (Hoff, 2003).

There was no effect of age at intervention or communication mode on psychosocial outcomes for children with HAs at 5 years of age. Similar to children with CIs, the current cohort received intervention for hearing loss much earlier compared with previous studies. Consistent with findings from a systematic review of psychopathology risk factors (Theunissen et al., 2014c), the current study also found no effect of severity of hearing loss on psychosocial outcomes in children with HAs after controlling for other factors.

When language and functional auditory behavior scores were added to the regression model for children with HAs, the proportion of explained variance in psychosocial outcomes increased significantly by 8.5%. Both language and functional auditory behavior were significantly associated with global psychosocial outcomes for children with HAs. The findings support previous literature showing that the development of language and the ability to listen and communicate in various everyday environments are the strongest and most direct mediators of psychosocial functioning (Barker et al., 2009; Batten et al., 2014; Stevenson et al., 2010). Interestingly, the PEACH had a slightly larger effect size, indicating that good language alone may not be enough to improve psychosocial outcomes if the ability to communicate and listen at a functional level is poor. Fellinger et al. (2009) found that DHH children who had difficulties making themselves understood at home were 9.61 times more likely to have a lifetime diagnosis of a mental health disorder. The findings emphasize the importance of *functional* auditory and communicative behavior for healthy psychosocial development in both children with HAs and children with CIs, and suggest once again that the PEACH may be a potential screener for early psychosocial problems in DHH children.

### Future Directions and Limitations

Although a limitation of this study is its reliance on parent report for assessing child outcomes, this method is typically used for identifying psychosocial problems in young children. Furthermore, it is not known whether parents who completed the questionnaire in their non-native language had difficulties answering questions. However, the present study offered translated versions of the CDI and SDQ for families from NESB in an attempt to overcome any problems with language comprehension. Typically, clinical diagnoses of childhood emotional and behavioral disorders would require the child to show difficulties across multiple contexts (e.g., at home and school) and would use cross-informant ratings from parents and teachers (Achenbach, McConaughy, & Howell, 1987). The prospective nature of the LOCHI study will allow us to measure self-report when the children are older, and the data collected from teacher ratings will be reported in future studies.

An unavoidable limitation is the amount of missing data from the population-based study, and the use of multiple imputations in the analyses. While this method is one of the most commonly used approaches, not all variables were ‘missing at random’ thus introducing possible biases. Only independent variables were imputed and a large number (10) of imputations were created to reduce sampling variability. In addition, the CI group was much smaller than the HA group, reducing the power for this group in the regression analyses. However, a smaller number of predictor variables was used in this group, and the sample is still much larger than previous studies looking at psychosocial outcomes in children with CIs.

Finally, there was still a large proportion of unexplained variance, particularly for children with CIs, indicating that other variables may be contributing directly to psychosocial outcomes. There is some literature showing that factors such as parental mental health and resources (Hintermair, 2006; Kushalnagar et al., 2007), parental involvement (Calderon, 2000), the child’s executive function (Hintermair, 2013), motor skills (Fellinger, Holzinger, Aigner, Beitel, & Fellinger, 2015), and theory of mind ability (Peterson, Slaughter, Moore, & Wellman, 2016) are linked to psychosocial outcomes. Other factors, including the abovementioned, could be examined in future research.

## Conclusions

In conclusion, on average, 5-year-old DHH children with CIs or HAs did not show significant emotional or behavioral difficulties compared with norms (SDQ), although they did score more than 1*SD* below the norm on social skills development (CDI). For children with similar levels of hearing loss, children with HAs were rated as having significantly more behavioral problems compared with children with CIs. Some differences were seen in regards to the factors influencing global psychosocial functioning for the two device groups. For children with CIs, presence of additional disabilities and functional auditory behavior were significantly associated with global psychosocial outcomes, whereas non-verbal cognitive ability, language, and functional auditory behavior were significant factors for children with HAs. The only consistent influential factor for both groups was functional auditory behavior. The findings have implications for developing interventions that focus on improving early social skills, and improving the ability to listen and communicate at a functional level (e.g., ability to listen in noise, detect subtle social cues, and engage in turn-taking or conversational skills) to enhance psychosocial well-being in children with HAs and CIs.
